# SEOM-GECP Clinical guidelines for diagnosis, treatment and follow-up of small-cell lung cancer (SCLC) (2022)

**DOI:** 10.1007/s12094-023-03216-3

**Published:** 2023-07-07

**Authors:** Rosario García-Campelo, Ivana Sullivan, Edurne Arriola, Amelia Insa, Oscar Juan Vidal, Patricia Cruz-Castellanos, Teresa Morán, Noemí Reguart, Jon Zugazagoitia, Manuel Dómine

**Affiliations:** 1 Department of Medical Oncology, Hospital Universitario A Coruña, Health Research Institute, INIBIC, A Coruña, Spain; 2 Department of Medical Oncology, Hospital de la Santa Creu i Sant Pau, IIB Sant Pau, Barcelona, Spain; 3 Department of Medical Oncology, Hospital del Mar-CIBERONC, Barcelona, Spain; 4 Departmert of Medical Oncology, Hospital Clínico de Valencia, Valencia, Spain; 5 Department of Medical Oncology, Hospital Universitari i Politécnic La Fe de Valencia, Valencia, Spain; 6 Department of Medical Oncology, Hospital Universitario La Paz, Madrid, Spain; 7 Department of Medical Oncology, Badalona Applied Research Group in Oncology, Catalan Institute of Oncology Badalona, Hospital Universitario Germans Trias i Pujol, Institut Germans Trias i Pujol, Barcelona, Spain; 8 Department of Medical Oncology, Hospital Clinic, Barcelona, Spain; 9 Department of Medical Oncology, Tumor Microenvironment and Immunotherapy Research Group, Hospital Universitario 12 de Octubre, Madrid, Health Research Institute Hospital Universitario 12 de Octubre (i+12), H12O-CNIO Lung Cancer Clinical Research Unit, Health Research Institute, CIBERONC, Madrid, Spain; 10 Department of Medical Oncology. Hospital, Universitario Fundación Jiménez Díaz, IIS-FJD, Oncohealth Institute, Universidad Autónoma de Madrid, Madrid, Spain; 11 Universitat Autònoma de Barcelona, Barcelona, Spain

**Keywords:** Small-cell lung cancer, Clinical practice guidelines, Diagnosis, Treatment, Follow-up

## Abstract

Small-cell lung cancer (SCLC) is a highly aggressive malignancy comprising approximately 15% of lung cancers. Only one-third of patients are diagnosed at limited-stage (LS). Surgical resection can be curative in early stages, followed by platinum–etoposide adjuvant therapy, although only a minority of patients with SCLC qualify for surgery. Concurrent chemo-radiotherapy is the standard of care for LS-SCLC that is not surgically resectable, followed by prophylactic cranial irradiation (PCI) for patients without progression. For extensive-stage (ES)-SCLC, a combination of platinum and etoposide has historically been a mainstay of treatment. Recently, the efficacy of programmed death-ligand 1 inhibitors combined with chemotherapy has become the new front-line standard of care for ES-SCLC. Emerging knowledge regarding SCLC biology, including genomic characterization and molecular subtyping, and new treatment approaches will potentially lead to advances in SCLC patient care.

## Incidence and epidemiology

SCLC accounts for approximately 15% of all lung cancer diagnoses [1] and has been designated an orphan disease by the European Medicines Agency (EMA) given its low prevalence of 1–5 per 10,000 people in the European community [2]. More than two-thirds of all cases are diagnosed in extensive-stage (ES), according to the classification of the Veterans Administration Lung Study Group (VALG) and equivalent to stage IVA/B of the 8^th^ edition of the American Joint Committee on Cancer (AJCC), while only a minority are diagnosed in limited-stage (LS), stage I–III of the 8^th^ edition AJCC [1]. SCLC has a high propensity to spread to the brain, with approximately 10–20% of patients presenting with brain metastases (BM) at the initial diagnosis and eventually up to 40%-50% developing BM during the course of their disease [3]. Some reported prognostic factors include performance status (PS), increased age, male gender, and numerous metastatic sites at baseline [4]. But certainly, prognosis is largely related to the stage of the disease and therapeutic approach: whereas LS disease is potentially curable, with 20–30% of patients alive at 5 years and median overall survival (mOS) ranging between 25 and 30 months [5], while patients with ES disease have poor mOS of 10–13 months and 5-year survival rates of < 5% [1, 6, 7]. Smoking is known to be the primary risk factor for SCLC [6], and therefore, smoking prevention or cessation are key strategies to diminish the clinical impact of the disease. Although SCLC can also appear in non-smokers, such cases are being reported in less than < 5% of all cases [8]. Risk factors in this population group are poorly characterized, but they could represent a genetically distinguished subtype of SCLC [9]. Other associated environmental risk factors are less common, but include exposure to chemicals, asbestos, or radon gas. In recent decades, SCLC trends have been declining in most developed countries, due to tobacco control policies and this trend is projected to continue in the coming years. This incidence decrease, however, is more marked in men than in women, with a gender incidence disparity that is narrowing and closing [10].

## Methodology

This guideline is based on systematic review of relevant published studies and with the consensus of ten treatment expert oncologists from Spanish Lung Cancer Group (SLCG) and Spanish Society of Medical Oncology (SEOM), and an external review panel of two experts designated by SEOM. The Infectious Diseases Society of America-US Public Health Service Grading System for Ranking Recommendations in Clinical Guidelines has been used to assign levels of evidence and grades of recommendation.

## Diagnosis and pathology

The pathological diagnosis of SCLC should be made using the World Health Organization (WHO) classification (IV, A) [11]. Histology is preferred over cytology (V, A). SCLC is a type of neuroendocrine tumor of the lung characterized by small cells with scant cytoplasm, poorly defined cell borders, fine granular nuclear chromatin, and absent or inconspicuous nucleoli. Immunohistochemistry is characterized by being positive for synaptophysin, chromogranin, and NCAM/CD56. Careful counting of mitoses is essential, as much as is the most important histologic criterion for distinguishing SCLC from typical and atypical carcinoids. The mitotic index in SCLC is high, at least 10 mitoses/2 mm^2^, with a Ki-67 proliferative index between 50 and 100%. Not predictive biomarker has been validated in clinical practice and PD-L1 testing is not routinely recommended.

## Staging

Initial evaluation must include an adequate anamnesis, including medical and smoking history, physical examination, and laboratory testing with complete blood count, biochemistry including liver enzymes, sodium, potassium, calcium, glucose, lactate dehydrogenase levels and renal function tests (V, A). In patients’ candidates for radical thoracic radiation, pulmonary function tests should be performed (V, B). For stage I–II SCLC patients eligible for surgical resection, invasive mediastinal staging is required. The presence of neurologic paraneoplastic syndromes that can be aggravated by immunotherapy must be ruled out (V, C).

Full staging includes computed tomography (CT) scan (with intravenous contrast) of the chest/abdomen and brain imaging by magnetic resonance imaging (MRI) or CT scan (if MRI is not possible) (III, A). The use of Fluorine-18 fluorodeoxyglucose positron emission tomography/CT (18F-FDG-PET/TC) is recommended in patients with LS to assist thoracic radiotherapy in localized tumor (III, A); for those patients with solitary metastasis, pathological confirmation is recommended to clarify the stage (III, C). Should there be direct or indirect data of infiltration of bone marrow, the study should be completed with bone marrow aspiration and biopsy (III, B).

The 8^th^ edition of the AJCC TNM staging system should be used to define prognosis and personalized treatment options better (I, A) (Table 1). The VALSG classification still appears in clinical trials. LS is defined as the tumor being confined to one hemithorax and regional lymph nodes (stage I–III; T any, N any, M0), that can be treated with definitive chemoradiation therapy. ES defines disease outside of the thorax or not accessible for radiation therapy (stage IV; T any, N any, M1a/b/c; or T3-4 due to multiple lung nodules) [12].

**Table 1:** TNM classification (AJCC 8^th^edition)

T	N	M	AJCC stage
Tx	N0	M0	Occult
Tis	N0	M0	0
T1a	N0	M0	IA1
T1b	N0	M0	IA2
T1c	N0	M0	IA3
T2a	N0	M0	IB
T2b	N0	M0	IIA
T1a–T2b	N1	M0	IIB
T3	N0	M0	IIB
T3	N1	M0	IIIA
T4	N0/N1	M0	IIIA
T1a–T2b	N2	M0	IIIA
T3–T4	N2	M0	IIIB
T1a–T2b	N3	M0	IIIB
T3–T4	N3	M0	IIIC
Any T	Any N	M1a/M1b	IVA
Any T	Any N	M1c	IVB

## Management of LS-SCLC

LS-SCLC represents a small proportion of new SCLC diagnoses and 80–90% of the cases achieve a 25–30-month mOS and a 20–30% 5-year survival rate with multimodal treatment. Treatment decisions should be made carefully by a multidisciplinary tumor board. See Table 2. 

### Stage I–IIA (T1-T2, N0, M0): role of surgery, chemotherapy (ChT), radiotherapy (RT), and prophylactic cranial irradiation (PCI)

Treatment recommendations for stages I–IIA (T1-2, N0, M0), which represent fewer than 5% of all SCLC, include surgery after mediastinal staging, that should be performed by either surgery (mediastinoscopy, mediastinotomy, and video-assisted thoracoscopy) or endobronchial or oesophageal ultrasound-guided biopsy (IV, A). Only a minority of patients with SCLC qualify for surgical resection Lobectomy with a systematic lymph-node dissection is the preferred surgical procedure (II, A). The aim of surgery should be complete resection (III, A). Sub-lobar resections are not recommended (IV, E) [13]. Data from a systematic review performed in 2017 failed to support the role of surgery in patients with stage I–III SCLC [14]. However, conclusions were limited by the quality and the obsolescence of the results. More recent studies have proven that surgery could improve survival rates when SCLC is diagnosed early. Adjuvant combination of cisplatin plus etoposide is recommended as systemic treatment in stage I–IIA (IV, A).

There is consensus on postoperative concurrent ChT and RT (cCRT) after R1 and R2 resection (IV, A) [13]. There is not a randomized study for node negative stages I or II SCLC, who are medically inoperable. In patients with surgical contraindication or refusing surgery, either stereotactic body radiation therapy (SBRT) [15] or conventional fractionation is recommended (mediastinal staging is required) and ChT should be added if medically tolerable. SBRT is less suitable for ultracentral tumors (III, A). There is no evidence for PCI recommendation in surgically resected early stage SCLC. The meta-analysis of five retrospective studies showed that PCI was associated with a survival advantage in stage II–III SCLC patients who underwent surgery. No survival benefit was achieved in stage I–IIA (T1-2, N0, M0) patients [16]. Therefore, PCI is not recommended in this subgroup of patients (II, E).

### Stage IIB–IIIC (T1–T4, N0-3, M0): role of ChT and RT, sequence, timing, volume target, PCI, and consolidation treatment

Treatment recommendation for such stages (T1–T4, N0–N3, M0) is cCRT (I, A) followed by PCI (I, A). A combination of cisplatin plus etoposide is the recommended ChT regimen (I, A) [5, 17]. Nevertheless, the efficacy of cisplatin *versus* carboplatin containing regimens has long remained controversial. A meta-analysis confirmed the survival benefit for regimens including cisplatin, etoposide, or both over other combinations [18], while other meta-analysis revealed no differences [19]. As expected, the toxicity profile differed in both treatments. ChT should be recommended up to four cycles (II, B) [20]. Carboplatin should be reserved only when cisplatin is contraindicated (II, A) [19].

ChT dose reduction should be avoided, especially during the first two cycles, given that high initial doses of drugs may lead to a significantly improve long-term survival in LS-SCLC (II, B) [21].

The use of concurrent thoracic RT (TRT) is based on two randomized trials [5, 17]. The optimal dose and fractionation in LS-SCLC is 45 Gy delivered in 30 twice-daily fractions of 150 cGy over 3 weeks (I, A). The CONVERT trial was the first randomized clinical trial (RCT) to provide outcomes data of patients staged with PET-CT using the TNM classification and treated with modern RT techniques. OS did not differ significantly between the two groups (twice or once-daily RT). Toxicity was much lower than previously reported in the literature. There was no intergroup difference in respect to grade 3–4 esophagitis or grade 3–4 radiation pneumonitis. While CONVERT was designed to demonstrate superiority of once-daily RT, it turns out that 45 Gy delivered in 30, twice-daily fractions of 150 cGy over 3 weeks should remain the standard treatment in this patient population (I, A). For patients unable to undergo twice-daily treatment, daily RT of 60–70 Gy is an acceptable alternative (II, A) [5].

When administering cCRT, earlier TRT is superior to delayed RT and should start with ChT cycle 1 or 2. Meta-analyses assessing TRT timing suggest benefit from early TRT with certain caveats. The most comprehensive report, published in 2016, failed to detect an OS benefit for earlier or shorter TRT when data from nine studies including 2305 patients were analyzed with median 10 years of follow-up. Nonetheless, hazard ratio (HR) in the individual patient data meta-analysis favored earlier/shorter RT in trials in which subjects received ChT without a dose reduction or delay (II, A) [22].

When the patient PS or dose to the organs at risk does not allow for early administration of TRT, it may be postponed until the start of the third ChT cycle (II, B) [23]. Sequential CRT is an option for individuals who are not considered eligible for cCRT due to poor PS, comorbidities, and/or disease volume (V, B) [24].

For cases of LS-SCLC, RT of the affected field is recommended as the standard of care (defined as PET-avid fluorodeoxyglucose, enlarged on CT, and/or biopsy-positive) (III, A) [25].

For tumors that shrink with ChT in patients with LS-SCLC, treating all involved nodal stations (at the time of diagnosis) and post-ChT lung parenchymal tumor is recommended (III, A) [14].

PCI significantly decreases the risk of symptomatic brain metastases and increases OS in individuals with non-metastatic SCLC. PCI is currently offered to patients who respond to initial cCRT and have a PS of 0–1. The recommended dose is 25 Gy in 10 daily fractions (I, A) [26, 27]. The evidence supporting PCI is not as clear in patients with a post-cCRT PS of 2, in patients > 70 years of age and in those with pre-existing neurological conditions. In such cases, a shared decision process should be encouraged (IV, B).

Neurotoxicity of whole-brain radiation (WBRT) with neurocognitive decline is feared the most. Hippocampal avoidance (HA) techniques have been developed in an attempt to minimize these cognitive risks. Two similar modestly sized phase III trials comparing HA-PCI to standard PCI have yielded conflicting outcomes [28, 29]. The two trials were similar in size, used the same RT dose, and allowed enrollment of patients with either LS or ES. However, based on the improved cognitive outcomes of the GOECP-SEOR trial and the safety of HA-PCI with regards to survival and control of BM on both, the NKI and GOECP-SEOR trials, it is reasonable to offer HA-PCI as an alternative to standard PCI (II, B).

Mirroring consolidation therapy results in locally advanced non-small-cell lung cancer (NSCLC), several clinical trials have addressed the potential benefit of immune checkpoint inhibitors (ICIs) as maintenance therapy after CRT in LS-SCLC patients. The STIMULI trial has tested consolidation combination with nivolumab and ipilimumab [30]. No improvement in progression-free survival (PFS) has been demonstrated and grade ≥ 3 adverse events occurred in 62% and 25% in the experimental and control arms, respectively.

**Fig. 1:**
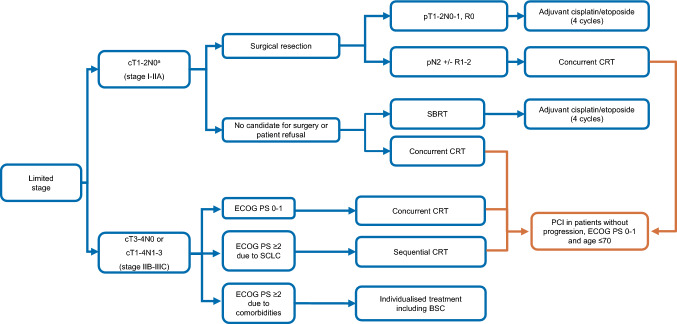
Treatment algorithm for limited-stage SCLC. *BSC* best supportive care, *CRT* chemo-radiotherapy, *ECOG* Eastern cooperative oncology group, *LN* lymph node, *PCI* prophylactic cranial irradiation, *PS* performance status, *RT* radiotherapy, *SBRT* stereotactic body radiation therapy, *SCLC* small-cell lung cancer; ^a^Pathological mediastinal staging negative

Consolidation therapy following CRT is under investigation in a phase III trial of durvalumab with or without tremelimumab (ADRIATIC trial, NCT NCT03703297). Ongoing phase II clinical trials are examining post-CRT consolidation therapy with atezolizumab (ACHILES trial, NCT03540420) and atezolizumab combined with CRT followed by consolidation atezolizumab (NCT03811002). Pembrolizumab and cCRT in the context of LS-SCLC are being studied in a phase I trial and in the KEYLINK-013, a phase III study of pembrolizumab in combination with cCRT followed by pembrolizumab with or without olaparib as consolidation (NCT04624204). While definitive efficacy results are awaited, these clinical trials have demonstrated the feasibility of incorporating such a strategy in the therapeutic scenario of LS-SCLC. Treatment algorithm for LS-SCLC is shown in Fig. 1.

## Management of ES-SCLC

### First-line systemic treatment

Immunotherapy (IO), particularly ICIs targeting programmed death-ligand 1 (PD-L1), has been added to the standard ChT in the first-line treatment of SCLC. Two phase III RCTs, IMpower-133 and CASPIAN [31, 32], have demonstrated the benefit of adding atezolizumab and durvalumab, respectively, to the platinum and etoposide doublet. In both trials, patients with ES-SCLC, Eastern Cooperative Group (ECOG), PS 0–1 were included. Asymptomatic treated or untreated brain metastases were allowed. See Table 2.

IMpower-133 evaluated the efficacy and safety of atezolizumab or placebo, added to carboplatin plus etoposide for four cycles, followed by maintenance atezolizumab or placebo until progression, unacceptable toxicity, or patient withdrawal. The two co-primary endpoints, OS and PFS, were met. Median OS was 12.3 months for atezolizumab vs 10.3 months for placebo (HR 0.70; 95% confidence interval [CI] 0.54–0.91; *p* = 0.0069). At 24 months, with a median follow-up of 22.9 months, 22% of the atezolizumab group were alive compared to 16.8% in the placebo group. Median PFS was 5.2 months for atezolizumab *versus* 4.3 months for placebo (HR 0.77; 95% CI 0.62–0.96; *p* = 0.017). OS and PFS improvements were consistent across patient subgroups.

CASPIAN assessed the efficacy and safety of adding durvalumab and/or tremelimumab in subjects with treatment naïve, ES-SCLC. This was a three-arm trial and patients were randomized 1:1:1 to receive either platinum plus etoposide and durvalumab, with or without tremelimumab, for four cycles, followed by maintenance durvalumab; ChT for up to six cycles in the control arm was allowed. In the comparison between ChT alone and the durvalumab arm, mOS of 12.9 months was observed for durvalumab plus ChT versus 10.5 months for ChtT alone (HR 0.75; 95% CI 0.62–0.91; *p* = 0.0032). At 24 months, 22.9% of the participants were alive in the durvalumab arm compared to 13.9% in the ChT alone group. Updated 3-year OS have been reported affirming that 17.6% vs. 5.8% subjects were alive at 3 years for the durvalumab and ChT alone arms, respectively [7]. Notably, neither study allowed cRT. As for PCI, the IMpower-133 and the standard ChT-only arm in CASPIAN did, but only a limited number of patients, around 10% received PCI.

To identify the subjects who benefit most, both studies have performed exploratory analyses to assess the role of clinical characteristics and biomarkers (PD-L1 and tumor mutational burden) as predictive biomarkers. Unfortunately, no patient or tumor characteristics proved to have a predictive role in these analyses.

Therefore, all patients with advanced SCLC with ECOG PS 0–1, controlled BM, and no previous treatment should be offered the combination of standard ChT plus atezolizumab or durvalumab (I,A).

A third phase III clinical trial exploring the addition of pembrolizumab in the same setting, the KEYNOTE 604, displayed numerically similar results but did not meet the statistical threshold for significance in OS [33]; consequently, this regimen has not been approved for clinical use.

The results of these trials are consistent in exhibiting a significant OS benefit. A recent meta-analysis of trials using ICIs evidenced that the addition of ICIs to platinum–etoposide yielded OS (HR 0.85; 95% CI 0.79–0.96) and PFS benefits (HR 0.78; 95% CI 0.72–0.83).

Recent phase III trials testing adebrelimab (anti-PD-L1 Capstone-1 Trial) and serplulimab (anti-PD-1, ASTRUM-005) and with a majority of Asian population confirm the results of the previous trials [34, 35].

**Table 2:** Recommended systemic regimens for SCLC

Systemic regimens for LS-SCLC: ChT should be administered up to a maximum of 4–6 cycles
Preferred regimens
Cisplatin 75 mg/m^2^ day 1 and etoposide 100 mg/m^2^ days 1–3, every 21 days alternative regimens
Cisplatin 25 mg/m^2^ day 1–3 and etoposide 100 mg/m^2^ days 1–3, every 21 days
Carboplatin AUC 5–6 day 1 and etoposide 100 mg/m^2^ days 1–3, every 21 days
Systemic regimens for ES-SCLC first-line
Preferred regimens: combination of chemotherapy (ChT) + immunotherapy (IO)
Carboplatin AUC 5 day 1 and etoposide 100 mg/m^2^ days 1–3 and atezolizumab 1200 mg day 1 every 21 days × 4 cycles followed by maintenance atezolizumab 1200 mg day 1, every 21 days (I, A)
Carboplatin AUC 6 day 1 and etoposide 100 mg/m^2^ days 1, 2, 3 and durvalumab 1500 mg day 1 every 21 days × 4 cycles followed by maintenance durvalumab 1500 mg day 1 every 28 days (I, A)
Cisplatin 75 mg/m^2^ day 1 and etoposide 100 mg/m^2^ days 1–3 and durvalumab 1500 mg day 1 every 21 days × 4 cycles followed by maintenance durvalumab 1500 mg day 1 every 28 days (I, A)
Recommended regimens of ChT (4–6 cycles)
Preferred Regimen
Cisplatin 75 mg/m^2^ day 1 and etoposide 100 mg/m^2^ days 1–3, every 21 days (I, A)
Alternative regimens
Carboplatin AUC 5 day 1 and etoposide 100 mg/m^2^ days 1–3, every 21 days (I, A)
Optional ChT Regimens (II, B)
Carboplatin AUC 5 day 1 and irinotecan 50 mg/m^2^ days 1, 8, 15 every 28 days
Cisplatin 60 mg/m^2^ day 1 and irinotecan 60 mg/m^2^ days 1, 8, 15 every 28 days
Cisplatin 30 mg/m^2^ days 1, 8 and irinotecan 65 mg/m^2^ days 1, 8 every 21 days
Systemic regimens for second line
Relapse ≤ 3 months
Preferred Regimens
Clinical trial (recommended)
Topotecan IV 1.5 mg/m^2^ days 1–5 every 3 weeks (I, A)
Cyclophosphamide/doxorubicin/vincristine (CAV)
Lurbinectedin
Other options
Paclitaxel
Irinotecan
Relapse ≥ 3 months
Preferred regimen
Reinduction with platinum–etoposide (II, B)

### Thoracic RT for ES-SCLC

The role of TRT in the context of ES-SCLC has been addressed in randomized clinical trials [36–38]. The CREST trial randomly assigned patients to TRT or best supportive care (BSC) found no statistical difference regarding the primary endpoint of 1-year survival, yet a post hoc analysis pointed toward improve 2-year survival with radiotherapy, with a low rate of toxicity. OS and PFS were significantly better in patients with fewer than three distant metastases. Improved OS was confirmed in one meta-analysis, although an updated meta-analysis did not show benefit in OS and only significant improvements in PFS and reduction in thoracic failures [39, 40]. Thus, in patients with PS of 0–2 who achieve a response after ChT, TRT to the residual tumor and lymph nodes (30 Gy/10 fractions) is a treatment option (II, B).

One important unanswered question in the metastatic setting is the integration of TRT with the combined treatment of ChT–IO. Given that palliative doses are expected to have limited toxicity, the task force’s expert opinion is that 30 Gy of TRT can be safely administered to these patients with residual thoracic disease after the completion of ChT–IO (V, C).

### PCI for ES-SCLC

The role of PCI in the setting of metastatic disease is controversial, since a European phase III study suggested a survival benefit [41]. However, a Japanese phase III study found that, with MRI surveillance, PCI did not offer a benefit for patients with metastatic SCLC [42]. Clinical trials to solve this controversy are ongoing. Therefore, for patients who can adhere to the schedule, MRI surveillance can be contemplated as an alternative to PCI. Among patients with ES-SCLC who elect to have PCI, data to support the use of 25 Gy in ten fractions to control the disease with acceptable neurotoxicity (II, B).

**Fig. 2:**
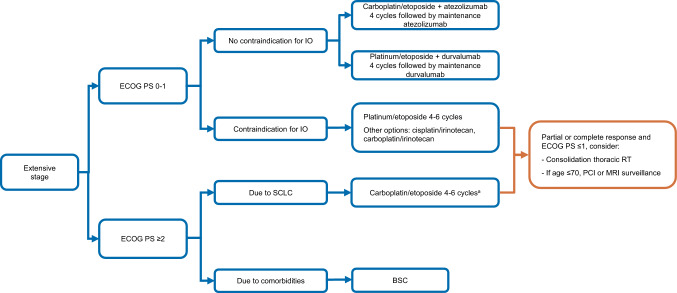
Treatment algorithm for extensive-stage SCLC. *BSC* best supportive care, *ECOG* Eastern cooperative oncology group, *G-CSF* granulocyte colony-stimulating factor, *IO* immunotherapy, *MRI* magnetic resonance imaging, *PCI* prophylactic cranial irradiation, *PS* performance status, *RT* radiotherapy, *SCLC* small-cell lung cancer; ^a^Consider the use of G-CSF in patients ≥ 70 years

The IMpower-133 trial did not mandate PCI and only 11% of participants received PCI. Those who receive combined ChT–IO are now living longer and the benefit of adding PCI has yet to be determined. Additional research is needed regarding the indication of this strategy (V, C). Treatment algorithm for ES-SCLC is shown in Fig. 2.

### Radiotherapy for BM

Some studies have reported encouraging outcomes with radiosurgery for recurrent BM in SCLC after PCI or WBRT [43]. Additionally, there is a growing body of literature that endorsed the viability of first-line radiosurgery in selected cases of SCLC in the absence of prior PCI or WBRT that have reported low rates of neurologic mortality and outcomes comparable to those of patients with NSCLC managed with the same approach [44]. The FIRE-SCLC cohort study was a large multicentre, retrospective analysis that yielded encouraging results of radiosurgery for BM, particularly among subjects with a single metastasis [45]. Radiosurgery may be a treatment option for carefully selected patients with SCLC (IV, C).

## Relapsed SCLC: second and further lines treatment options

Responses to front-line ChT–IO in ES-SCLC do not last in most patients. In the relapse setting, three clinical scenarios have been defined based on prior sensitivity to front-line platinum-based ChT: (1) sensitive disease, defined by disease progression after 3 months last dose of platinum; (2) resistant disease (relapse occurring within 3 months); and (3) refractory disease (progressive disease as best response to front-line therapy). In a pooled analysis of 21 studies of relapsed second-line SCLC, sensitive patients had a significantly higher response rate (RR) (27.7%) and longer mOS (7.7 months) as compared to refractory patients (14.8% and 5.4 months) [46].

All cases of relapsed SCLC should be assessed for inclusion in a clinical trial. The treatment decision should be based on PS, comorbidities, toxicity, and disease-free interval from prior therapy.

For sensitive patients whose disease has relapsed or progress after 3 months after completion of first-line treatment, rechallenge with platinum–etoposide ChT is recommended (II, B).

Topotecan is the only drug currently approved in Europe for second-line SCLC, based in two randomized phase III trials [47, 48]. Despite its modest efficacy, RRs not exceeding 25% and mOS usually of 6–9 months, no other therapy has shown survival benefit to topotecan in any of the multiple randomized phase III trials in the relapsed setting. Oral or i.v. topotecan is recommended for patients with platinum-resistant or -sensitive relapse (I, A), an alternative is the combination of cyclophosphamide, doxorubicin and vincristine (CAV) (II, B). Beyond topotecan, other ChT agents, such as irinotecan or paclitaxel, are commonly used to treat relapsed SCLC. These drugs offer modest, yet somewhat comparable efficacy to topotecan and are generally associated with a better tolerability [49].

**Fig. 3:**
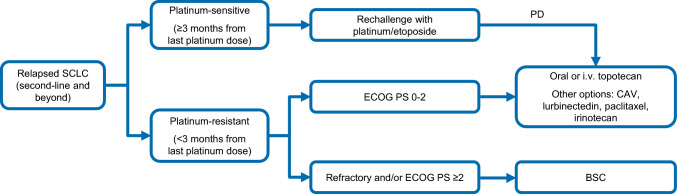
Treatment algorithm for relapsed SCLC. *BSC* best supportive care, *CAV* cyclophosphamide/doxorubicin/vincristine, *ECOG* Eastern cooperative oncology group, *i.v.* intravenous, *PD* progressive disease, *PS* performance status

Lurbinectedin is a cytotoxic ChT agent that appears to selectively inhibit active transcription in tumor cells and tumor-associated macrophages. In a phase II study in relapsed SCLC, lurbinectedin monotherapy revealed a 35% RR, and mPFS and mOS were 3.5 months and 9.3 months, respectively [50]. Based on the results of this study, lurbinectedin monotherapy was granted accelerated approval by the FDA and orphan drug designation by the EMA for the treatment of relapsed SCLC. Nevertheless, despite this promising activity as single agent, the randomized phase III ATLANTIS trial that compared lurbinectedin plus doxorubicin with topotecan or CAV has failed to meet the prespecified superiority endpoint of OS [51]. Treatment algorithm for relapsed SCLC is shown in Fig. 3.

ICIs have demonstrated modest activity in relapsed patients (RR 10–15%) in phase I/II trials [52, 53]. The only phase III study comparing nivolumab vs standard ChT (CheckMate-331) was negative [54]. Attempts to identify predictive biomarkers in the context of relapsed disease have been elusive, and PD-1 axis inhibitors have not been approved and cannot be generally recommended as monotherapy for relapsed SCLC.

Other immunotherapy strategies as, tarlatamab (AMG757), a DLL-3/CD3 bi-specific T-cell engager (BiTE) exhibited an encouraging RR of 23.4% with a and most notably a median duration of response of 12.3 months in an ongoing phase I study that included heavily pre-treated SCLC patient [55]. Multiple novel biologic agents are under rigorous investigation in relapsed SCLC. Among non-immune agents, drugs targeting DNA Damage Response (DDR) pathways including PARP or ATR inhibitors have yielded clinical responses in early phase studies and deserve further clinical investigation in larger studies [56, 57]. In addition, novel antibody drug conjugates (ADCs), such as ifinatamab deruxtecan (a B7-H3-directed ADC), have shown encouraging early signs of clinical activity in a small cohort of SCLC patients enrolled in a phase 1/2 first in human trial [58]. Nevertheless, larger confirmatory trials are needed before drawing definitive conclusions on the potential of these new agents to impact the clinical care of patients with relapsed SCLC. New classification of SCLC subtypes defined by distinct gene expression profiles could help us in designing new clinical trials.

## Elderly and frail patients

Over the last 30 years, the proportion of elderly patients (≥ 70 years of age) with SCLC has doubled. Elderly patients are often frail, under-represented in clinical trials, and present multiple comorbidities that make selecting optimal treatment even more challenging. Geriatric assessment tools are highly recommended for individualized treatment choice. For LS-SCLC, the potential of achieving long-term survival justified a more aggressive approach in elderly patients with good PS. cCRT could be considered the standard treatment for fit elderly patients (IV, B), conferring similar RR and OS, in elderly and young patients, although greater toxicity [59]. Unfit patients ineligible for cCRT may be considered for sequential CRT, with the option of a more conservative RT approach treating the post-ChT primary tumor volume and the pre-ChT nodal volume (II, C) [60]. For elderly ES-SCLC patients, carboplatin/etoposide regimen is preferred than cisplatin/etoposide (I, B) [19]. Atezolizumab and durvalumab combined with ChT are recommended as the first-line treatment for elderly patients with ES-SCLC (I, B) [31, 32]. Survival benefit results in subgroup analysis according to age (cut-off 65 years) are contradictory. While in the CASPIAN trial with durvalumab, the OS benefit appears to be limited to patients ≤ 65 years (HR 0.72; 95% CI 0.56–0.92) compared to patients ≥ 65 years (HR 0.84; 95% CI 0.62–1.12), in the IMpower-133 trial, the benefit with atezolizumab was higher in patients ≥ 65 years (HR 0.53; 95% CI 0.36–0.77) than in those aged ≤ 65 years (HR 0.92; 95% CI 0.64–1.32). Patients with PS ≥ 2 were not included in these trials and the use of ChT–IO upfront has not been established.

PCI was associated with an increased risk of neuropsychological impairment in older patients (age ≥ 60 years). Active CNS surveillance other than PCI is preferred in older patients with ES-SCLC (I, A) [42]. In LS-SCLC, although older meta-analysis seems to show similar benefit of PCI in elderly patients than in young patients [26], a shared decision process to discuss the risk and benefits of PCI over close surveillance is recommended.

## Drugs reducing ChT-induced myelosuppression

In SCLC, dose intensity is of paramount importance to achieve better outcomes; therefore, ChT dose reductions should be avoided.

For LS, the use of prophylactic granulocyte or granulocyte–macrophage colony-stimulating factors (G/GM-CSF) has demonstrated to be a safe supportive measure, with no detrimental effect on survival (II, B).

For ES, the use of G/GM-CSF is a treatment option to prevent hematologic toxicity (II, B). Trilaciclib added to platinum–etoposide has demonstrated a myelopreservation across multiple hematopoietic lineages leading to less supportive care interventions, dose reductions, and improved safety [61]. Trilaciclib has been approved for the FDA and included in NCCN guidelines as an option to reduce the frequency of to decrease the incidence of ChT-induced myelosuppression in ES-SCLC patients receiving ChT (II, A).

### Follow-up

For LS-SCLC, after treatment completion, a CT scan is recommended every 3 months for 2 years, then every 6 months during year 3, and then annually (V, C). For ES, after completing initial or subsequent therapy, CT scans every 2–3 months are recommended (V, C) [42]. MRI (preferred) or CT brain with contrast every 3 months during the first year, then every 6 months thereafter are recommended in patients who did not undergo PCI. Table 3 summarizes the recommendations.

**Table 3:** Summary of recommendations

Pathological diagnosis and staging	Pathological diagnosis of SCLC should be made using the World Health Organization classification Initial evaluation must include adequate anamnesis, medical/smoking histories, physical examination, complete blood count, and biochemistry, including liver enzymes, sodium, potassium, calcium, glucose, lactate dehydrogenase levels, and renal function test (V, A) Lung function tests in patients candidate to TRT (V, B) The presence of neurologic paraneoplastic syndromes that can be aggravated by immunotherapy must be ruled out (V, C) Full staging includes: CT scan with intravenous contrast of the chest/abdomen, MRI (preferred), or CT scan (with intravenous contrast) for brain imaging (III, A) 18F-FDG-PET/TC scan is recommended in localized disease to assist to thoracic radiotherapy (III, A). In patients with a solitary metastasis, its pathological confirmation is recommended (III, C) Bone marrow aspiration or biopsy is recommended if direct or indirect data of bone marrow infiltration (III, B) 8th edition of the TNM staging system according to the AJCC should be used (Table 1) (I, A). Combined use of TNM and VA classification is appropriate
Management of limited Stage I–IIA (T1–T2, N0, M0)	Surgery should be recommended in patients with clinical stages I and II (cT1-2N0) (III, B) Lobectomy with a systematic lymph-node dissection is the preferred surgical procedure after mediastinal staging (II, A) ChT and TRT (IV, A) concurrent (preferred) or sequentially (IV, A) should be recommended in patients with R0 pN1–pN2 or R1–R2 after surgery Patients with N0 disease should be recommended adjuvant chemotherapy (IV, A) SBRT (≥ 50 Gy) represents an alternative for patients with stage I–IIA SCLC with surgical contraindication or refusing surgery. After completion of SBRT patients should receive four cycles of adjuvant chemotherapy (III, A) PCI is not recommended in this subgroup of patients (II, E)
Management of limited-stage IIB–IIIC (T3–4, N0 M0; T1–4, N1–3, M0)	Patients should be treated with concurrent ChT and TRT (I, A) The recommended ChT is the combination of 4 cycles of cisplatin–etoposide (I, A). Carboplatin could replace cisplatin when contraindication (II, A) ChT dose reductions should be avoided, especially during the first two cycles of treatment (II, B) The use of G/GM-CSF is safe, when clinically indicated (II, B) 45 Gy with twice-daily fraction (I, A) or 60–70 Gy (II, A); with once-daily fraction are accepted treatments. Either of them should be administered concomitantly to systemic therapy (II, A) RT should be started as early as with the 1st or 2nd course of ChT (II, A) PCI (25 Gy in ten daily fractions) should be administered after CRT in patients without progression (I, A) Hippocampal avoidance PCI is an alternative option to PCI (II, B)
Management of extensive-stage (any T, any N, M1a, b, c): first-line treatment	The recommended first-line treatment is the use of platinum–etoposide + IO (I, A) Atezolizumab–carboplatin–etoposide 4 cycles followed by maintenance atezolizumab Durvalumab + carboplatin or cisplatin–etoposide 4 cycles followed by maintenance durvalumab If no candidate to receive IO, the recommended treatment is chemotherapy 4 cycles of cisplatin–etoposide (I, A). Carboplatin could replace cisplatin when contraindicated (I, B) Alternative regimens are cisplatin–irinotecan, carboplatin– irinotecan (II, B)
Management of extensive-stage (any T, any N, M1a, b, c): radiotherapy	Consolidative thoracic radiation to the residual tumor and lymph nodes (30 Gy/10 fractions) in selected patients who achieved a response to ChT is a treatment option (II, B) PCI (25 Gy) should be evaluated in patients with good PS who achieve a response (II, B) An alternative to PCI in patients without brain metastases on brain MRI after ChT is follow up with regular brain MRI omitting PCI (II, B) The benefit of adding PCI in patients receiving ChT–IO has yet to be determined (V, C)
Second-line treatment in ES-SCLC	Retreatment with platinum–etoposide is recommended for patients with sensitive relapse (platinum-free interval ≥ 3 months) (I, A) Single-agent topotecan is recommended for patients with refractory disease, resistant relapse, or in patients with sensitive relapse that are not candidates for platinum rechallenge (e.g., ECOG PS > 1, prior significant toxicity with doublet platinum-based ChT, or any other contraindication to receive platinum) (I, B) In this same situation, CAV (II, B), irinotecan (III, B) or weekly paclitaxel (III, C) are also reasonable treatment options Single-agent lurbinectedin is clinically active in relapsed SCLC, and it can be considered and recommended in patients with relapsed SCLC regardless of platinum-free interval (III, A). At the time of writing guideline document, lurbinectedin is FDA approved, has granted orphan drug designation by the EMA, but not authorized in Spain Single-agent PD-1 axis blockade is not generally recommended in unselected patients with relapsed SCLC (I, D)
Elderly and frail patients	In LS-SCLC, concurrent cCRT with modern technics could be a treatment option for fit and elderly patients (IV, B) Unfit patients ineligible for cCRT may be considered for sequential (II, C) For elderly ES-SCLC patients, carboplatin/etoposide is preferred than cisplatin/etoposide (I, B) In ES-SCLC, ChT–IO combination are recommended as first-line treatment (I, B) Shared decision process to indicate PCI over close surveillance is recommended in older patients with LS-SCLC Active CNS surveillance than PCI is preferred in older patients with ES-SCLC (I, A)
Follow-up	LS-SCLC: CT scan every 3 months the first year, every 6 months year 2–3 and after annually (V, C) ES-SCLC: CT scan every 2–3 months the first year, every 3 months year 2 and 3, every 6 months year 4–5 and then annually (V, C) MRI (preferred) or CT brain with contrast every 3 months during the first year, then every 6 months thereafter are recommended in patients who did not undergo PCI

## References

[1] Lung Cancer—Small Cell: Statistics. Available at https://www.cancernet/cancer-types/lung-cancer-small-cell/statistics. Accessed 15 Sep 2022

[2] EU/3/21/2415: Orphan designation for the treatment of small cell lung cancer. Available at https://www.emaeuropaeu/en/medicines/human/orphan-designations/eu-3-21-2415. Accessed 15 Sep 2022

[3] Li N, Chu Y, Song Q (2021). Brain metastasis in patients with small cell lung cancer. Int J Gen Med.

[4] Foster NR, Mandrekar SJ, Schild SE, Nelson GD, Rowland KM, Deming RL (2009). Prognostic factors differ by tumor stage for small cell lung cancer: a pooled analysis of North central cancer treatment group trials. Cancer.

[5] Faivre-Finn C, Snee M, Ashcroft L, Appel W, Barlesi F, Bhatnagar A (2017). Concurrent once-daily versus twice-daily chemoradiotherapy in patients with limited-stage small-cell lung cancer (CONVERT): an open-label, phase 3, randomised, superiority trial. Lancet Oncol.

[6] Franco F, Carcereny E, Guirado M, Ortega AL, Lopez-Castro R, Rodriguez-Abreu D (2021). Epidemiology, treatment, and survival in small cell lung cancer in Spain: data from the thoracic tumor registry. PLoS One.

[7] Paz-Ares L, Chen Y, Reinmuth N, Hotta K, Trukhin D, Statsenko G (2022). Durvalumab, with or without tremelimumab, plus platinum–etoposide in first-line treatment of extensive-stage small-cell lung cancer: 3-year overall survival update from CASPIAN. ESMO Open.

[8] Ou S-HI, Ziogas A, Zell JA (2009). Prognostic factors for survival in extensive stage small cell lung cancer (ED-SCLC): the importance of smoking history, socioeconomic and marital statuses, and ethnicity. J Thorac Oncol.

[9] Sivakumar S, Moore JA, Montesion M, Sharaf R, Lin DI, Fleishmann Z (2022). Integrative analysis of a large real-world cohort of small cell lung cancer identifies distinct genetic subtypes and insights into histological transformation. bioRxiv.

[10] Govindan R, Page N, Morgensztern D, Read W, Tierney R, Vlahiotis A (2006). Changing epidemiology of small-cell lung cancer in the United States over the last 30 years: analysis of the surveillance, epidemiologic, and end results database. J Clin Oncol.

[11] Travis WD, Brambilla E, Burke AP, Marx A, Nicholson AG (2015). WHO classification of tumours of the lung, pleura, thymus and heart Lyon: International agency for research on cancer. J Thorac Oncol.

[12] Amin MB, Greene FL, Byrd DR, Edge SB, Compton CC, Gershenwald JE (2016). American joint committee on cancer (AJCC) cancer staging manual.

[13] Ernani V, Ganti AK (2017). Surgery for limited-stage small cell lung cancer: ready for prime-time?. J Thorac Dis.

[14] Barnes H, See K, Barnett S, Manser R (2017). Surgery for limited-stage small-cell lung cancer. Cochrane Database Syst Rev.

[15] Verma V, Simone CB, Allen PK, Lin SH (2017). Outcomes of stereotactic body radiotherapy for T1–T2N0 small cell carcinoma according to addition of chemotherapy and prophylactic cranial irradiation: a multicenter analysis. Clin Lung Cancer.

[16] Yang Y, Zhang D, Zhou X, Bao W, Ji Y, Sheng L (2018). Prophylactic cranial irradiation in resected small cell lung cancer: a systematic review with meta-analysis. J Cancer.

[17] Turrisi AT, Kim K, Blum R, Sause WT, Livingston RB, Komaki R (1999). Twice-daily compared with once-daily thoracic radiotherapy in limited small-cell lung cancer treated concurrently with cisplatin and etoposide. N Engl J Med.

[18] Mascaux C, Paesmans M, Berghmans T, Branlen F, Lafitte JJ, Lemaitre F (2000). A systematic review of the role of etoposide and cisplatin in the chemotherapy of small cell lung cancer with methodology assessment and meta-analysis. Lung Cancer.

[19] Rossi A, Di Maio M, Chiodini P, Rudd RM, Okamoto H, Skarlos DV (2012). Carboplatin-or cisplatin-based chemotherapy in first-line treatment of small-cell lung cancer: the COCIS meta-analysis of individual patient data. J Clin Oncol.

[20] Veslemes M, Polyzos A, Latsi P, Dimitroulis J, Stamatiadis D, Dardoufas C (1998). Optimal duration of chemotherapy in small cell lung cancer: a randomized study of 4 versus 6 cycles of cisplatin-etoposide. J Chemother.

[21] Arriagada R, Le Chevalier T, Pignon JP, Riviere A, Monnet I, Tuchais C (1993). Initial chemotherapeutic doses and long-term survival in limited small-cell lung cancer. N Engl J Med.

[22] De Ruysscher D, Lueza B, Le Pechoux C, Johnson DH, O'Brien M, Murray N (2016). Impact of thoracic radiotherapy timing in limited-stage small-cell lung cancer: usefulness of the individual patient data meta-analysis. Ann Oncol.

[23] Sun JM, Ahn YC, Choi EK, Ahn MJ, Ahn JS, Lee SH (2013). Phase III trial of concurrent thoracic radiotherapy with either first- or third-cycle chemotherapy for limited-disease small-cell lung cancer. Ann Oncol.

[24] Dingemans AC, Fruh M, Ardizzoni A, Besse B, Faivre-Finn C, Hendriks LE (2021). Small-cell lung cancer: ESMO clinical practice guidelines for diagnosis, treatment and follow-up(). Ann Oncol.

[25] Colaco R, Sheikh H, Lorigan P, Blackhall F, Hulse P, Califano R (2012). Omitting elective nodal irradiation during thoracic irradiation in limited-stage small cell lung cancer–evidence from a phase II trial. Lung Cancer.

[26] Auperin A, Arriagada R, Pignon JP, Le Pechoux C, Gregor A, Stephens RJ (1999). Prophylactic cranial irradiation for patients with small-cell lung cancer in complete remission. Prophylactic cranial irradiation overview collaborative group. N Engl J Med.

[27] Le Pechoux C, Dunant A, Senan S, Wolfson A, Quoix E, Faivre-Finn C (2009). Standard-dose versus higher-dose prophylactic cranial irradiation (PCI) in patients with limited-stage small-cell lung cancer in complete remission after chemotherapy and thoracic radiotherapy (PCI 99–01, EORTC 22003–08004, RTOG 0212, and IFCT 99–01): a randomised clinical trial. Lancet Oncol.

[28] Belderbos JSA, De Ruysscher DKM, De Jaeger K, Koppe F, Lambrecht MLF, Lievens YN (2021). Phase 3 randomized trial of prophylactic cranial irradiation with or without hippocampus avoidance in SCLC (NCT01780675). J Thorac Oncol.

[29] de Dios NR, Counago F, Murcia-Mejia M, Rico-Oses M, Calvo-Crespo P, Samper P (2021). Randomized phase III trial of prophylactic cranial irradiation with or without hippocampal avoidance for small-cell lung cancer (PREMER): a GICOR-GOECP-SEOR study. J Clin Oncol.

[30] Peters S, Pujol JL, Dafni U, Dómine M, Popat S, Reck M, Andrade J, ETOP/IFCT 4-12 STIMULI Collaborators (2022). Consolidation nivolumab and ipilimumab versus observation in limited-disease small-cell lung cancer after chemo-radiotherapy—results from the randomised phase II ETOP/IFCT 4–12 STIMULI trial. Ann Oncol.

[31] Horn L, Mansfield AS, Szczęsna A, Havel L, Krzakowski M, Hochmair MJ, IMpower133 Study Group (2018). First-line atezolizumab plus chemotherapy in extensive-stage small-cell lung cancer. N Engl J Med.

[32] Paz-Ares L, Dvorkin M, Chen Y, Reinmuth N, Hotta K, Trukhin D, CASPIAN investigators (2019). Durvalumab plus platinum–etoposide versus platinum–etoposide in first-line treatment of extensive-stage small-cell lung cancer (CASPIAN): a randomised, controlled, open-label, phase 3 trial. Lancet.

[33] Rudin CM, Awad MM, Navarro A, Gottfried M, Peters S, Csőszi T, KEYNOTE-604 Investigators (2020). Pembrolizumab or placebo plus etoposide and platinum as first-line therapy for extensive-stage small-cell lung cancer: randomized, double-blind, phase III KEYNOTE-604 study. J Clin Oncol.

[34] Cheng Y, Han L, Wu L, Chen J, Sun H, Wen G (2022). Effect of First-line serplulimab vs placebo added to chemotherapy on survival in patients with extensive-stage small cell lung cancer: the ASTRUM-005 randomized clinical trial. JAMA.

[35] Wang J, Zhou C, Yao W, Wang Q, Min X, Chen G, CAPSTONE-1 Study Group (2022). Adebrelimab or placebo plus carboplatin and etoposide as first-line treatment for extensive-stage small-cell lung cancer (CAPSTONE-1): a multicentre, randomised, double-blind, placebo-controlled, phase 3 trial. Lancet Oncol.

[36] Jeremic B, Shibamoto Y, Nikolic N, Milicic B, Milisavljevic S, Dagovic A (1999). Role of radiation therapy in the combined-modality treatment of patients with extensive disease small-cell lung cancer: a randomized study. J Clin Oncol.

[37] Slotman BJ, van Tinteren H, Praag JO, Knegjens JL, El Sharouni SY, Hatton M (2015). Use of thoracic radiotherapy for extensive stage small-cell lung cancer: a phase 3 randomised controlled trial. Lancet.

[38] Slotman BJ, Faivre-Finn C, van Tinteren H, Keijser A, Praag J, Knegjens J (2017). Which patients with ES-SCLC are most likely to benefit from more aggressive radiotherapy: a secondary analysis of the phase III CREST trial. Lung Cancer.

[39] Palma DA, Warner A, Louie AV, Senan S, Slotman B, Rodrigues GB (2016). Thoracic radiotherapy for extensive stage small-cell lung cancer: a meta-analysis. Clin Lung Cancer.

[40] Rathod S, Jeremic B, Dubey A, Giuliani M, Bashir B, Chowdhury A (2019). Role of thoracic consolidation radiation in extensive stage small cell lung cancer: a systematic review and meta-analysis of randomised controlled trials. Eur J Cancer.

[41] Slotman B, Faivre-Finn C, Kramer G, Rankin E, Snee M, Hatton M (2007). Prophylactic cranial irradiation in extensive small-cell lung cancer. N Engl J Med.

[42] Takahashi T, Yamanaka T, Seto T, Harada H, Nokihara H, Saka H (2017). Prophylactic cranial irradiation versus observation in patients with extensive-disease small-cell lung cancer: a multicentre, randomised, open-label, phase 3 trial. Lancet Oncol.

[43] Bernhardt D, Bozorgmehr F, Adeberg S, Opfermann N, von Eiff D, Rieber J (2016). Outcome in patients with small cell lung cancer re-irradiated for brain metastases after prior prophylactic cranial irradiation. Lung Cancer.

[44] Wegner RE, Olson AC, Kondziolka D, Niranjan A, Lundsford LD, Flickinger JC (2011). Stereotactic radiosurgery for patients with brain metastases from small cell lung cancer. Int J Radiat Oncol Biol Phys.

[45] Rusthoven CG, Yamamoto M, Bernhardt D, Smith DE, Gao D, Serizawa T (2020). Evaluation of first-line radiosurgery vs whole-brain radiotherapy for small cell lung cancer brain metastases: the FIRE-SCLC Cohort Study. JAMA Oncol.

[46] Owonikoko TK, Behera M, Chen Z, Bhimani C, Curran WJ, Khuri FR (2012). A systematic analysis of efficacy of second-line chemotherapy in sensitive and refractory small-cell lung cancer. J Thorac Oncol.

[47] O’Brien MER, Ciuleanu TE, Tsekov H, Shparyk Y, Čučeviá B, Juhasz G (2006). Phase III trial comparing supportive care alone with supportive care with oral topotecan in patients with relapsed small-cell lung cancer. J Clin Oncol.

[48] von Pawel J, Schiller JH, Shepherd FA, Fields SZ, Kleisbauer JP, Chrysson NG (1999). Topotecan versus cyclophosphamide, doxorubicin, and vincristine for the treatment of recurrent small-cell lung cancer. J Clin Oncol.

[49] Zugazagoitia J, Paz-ares L (2022). Extensive-stage small-cell lung cancer: first-line and second-line treatment options. J Clin Oncol.

[50] Trigo J, Subbiah V, Besse B, Moreno V, López R, Sala MA (2020). Lurbinectedin as second-line treatment for patients with small-cell lung cancer: a single-arm, open-label, phase 2 basket trial. Lancet Oncol.

[51] Paz-Ares L, Ciuleanu T, Navarro A, Fulop A, Cousin S, Bonanno L (2021). PL02.03 lurbinectedin/doxorubicin versus CAV or topotecan in relapsed SCLC patients: phase III randomized ATLANTIS Trial. J Thorac Oncol.

[52] Antonia SJ, López-Martin JA, Bendell J, Ott PA, Taylor M, Eder JP (2016). Nivolumab alone and nivolumab plus ipilimumab in recurrent small-cell lung cancer (CheckMate 032): a multicentre, open-label, phase 1/2 trial. Lancet Oncol.

[53] Chung HC, Piha-Paul SA, Lopez-Martin J, Schellens JHM, Kao S, Miller WH (2020). Pembrolizumab after two or more lines of previous therapy in patients with recurrent or metastatic SCLC: results from the KEYNOTE-028 and KEYNOTE-158 studies. J Thorac Oncol.

[54] Spigel DR, Vicente D, Ciuleanu TE, Gettinger S, Peters S, Horn L (2021). Second-line nivolumab in relapsed small-cell lung cancer: CheckMate 331. Ann Oncol.

[55] Paz Ares L, Champiat S, Lai WV, Izumi H, Govindan R, Boyer M (2023). Tarlatamab, a first-in-class DLL3-targeted biespecific T-Cell engager, in recurrent small cell lung cancer: an open-label phase I study. J Clin Oncol.

[56] Pietanza MC, Waqar SN, Krug LM, Dowlati A, Hann CL, Chiappori A (2018). Randomized, double-blind, phase II study of temozolomide in combination with either veliparib or placebo in patients with relapsed-sensitive or refractory small-cell lung cancer. J Clin Oncol.

[57] Farago AF, Yeap BY, Stanzione M, Hung YP, Heist RS, Marcoux JP (2019). Combination olaparib and temozolomide in relapsed small-cell lung cancer. Cancer Discov.

[58] Doi T, Patel M, Falchook GS, Koyama T, Friedman CF, Piha-Poul S (2022). DS-7300 (B7-H3 DXd antibody-drug conjugate [ADC]) shows durable antitumor activity in advanced solid tumors: extended follow-up of a phase I/II study. Ann Oncol.

[59] Christodoulou M, Blackhall F, Mistry H, Leylek A, Knegjens J, Remouchamps V (2019). Compliance and outcome of elderly patients treated in the concurrent once-daily versus twice-daily radiotherapy (CONVERT) trial. J Thorac Oncol.

[60] Hu X, Bao Y, Jin XY, Neng ZH, Shi LJ, Zhang L (2020). Final report of a prospective randomized study on thoracic radiotherapy target volume for limited-stage small cell lung cancer with radiation dosimetric analyses. Cancer.

[61] Weiss J, Goldschmidt J, Andric Z, Dragnev KH, Gwaltney C, Skaltsa K (2021). Effects of trilaciclib on chemotherapy-induced myelosuppression and patient-reported outcomes in patients with extensive-stage small cell lung cancer: pooled results from three phase II randomized, double-blind, placebo-controlled studies. Clin Lung Cancer.

