# Correction to: SEOM-GECP Clinical guidelines for diagnosis, treatment and follow-up of small-cell lung cancer (SCLC) (2022)

**DOI:** 10.1007/s12094-023-03290-7

**Published:** 2023-08-09

**Authors:** Rosario García-Campelo, Ivana Sullivan, Edurne Arriola, Amelia Insa, Oscar Juan Vidal, Patricia Cruz-Castellanos, Teresa Morán, Noemí Reguart, Jon Zugazagoitia, Manuel Dómine

**Affiliations:** 1 Department of Medical Oncology, Hospital Universitario A Coruña, Health Research Institute, INIBIC, A Coruña, Spain; 2 Department of Medical Oncology, Hospital de la Santa Creu i Sant Pau, IIB Sant Pau, Barcelona, Spain; 3 Department of Medical Oncology, Hospital del Mar-CIBERONC, Barcelona, Spain; 4 Departmert of Medical Oncology, Hospital Clínico de Valencia, Valencia, Spain; 5 Department of Medical Oncology, Hospital Universitari i Politécnic La Fe de Valencia, Valencia, Spain; 6 Department of Medical Oncology, Hospital Universitario La Paz, Madrid, Spain; 7 Department of Medical Oncology, Badalona Applied Research Group in Oncology, Catalan Institute of Oncology Badalona, Hospital Universitario Germans Trias i Pujol, Institut Germans Trias i Pujol, Barcelona, Spain; 8 Department of Medical Oncology, Hospital Clinic, Barcelona, Spain; 9 Department of Medical Oncology, Tumor Microenvironment and Immunotherapy Research Group, Hospital Universitario 12 de Octubre, Madrid, Health Research Institute Hospital Universitario 12 de Octubre (i+12), H12O-CNIO Lung Cancer Clinical Research Unit, Health Research Institute, CIBERONC, Madrid, Spain; 10 Department of Medical Oncology. Hospital, Universitario Fundación Jiménez Díaz, IIS-FJD, Oncohealth Institute, Universidad Autónoma de Madrid, Madrid, Spain; 11 Universitat Autònoma de Barcelona, Barcelona, Spain

**Correction to: Clinical and Translational Oncology** 10.1007/s12094-023-03216-3

In Table 3 of this article, for the point "Second-line treatment in ES-SCLC", the statement "At the time of writing guideline document, lurbinectedin is FDA and EMA approved but not authorized in Spain" was incorrect but it should have been "At the time of writing guideline document, lurbinectedin is FDA approved, has granted orphan drug designation by the EMA, but not authorized in Spain." The corrected Table 3 is provided below.

**Table 3:** Summary of recommendations

Pathological diagnosis and staging	Pathological diagnosis of SCLC should be made using the World Health Organization classification Initial evaluation must include adequate anamnesis, medical/smoking histories, physical examination, complete blood count, and biochemistry, including liver enzymes, sodium, potassium, calcium, glucose, lactate dehydrogenase levels, and renal function test (V, A) Lung function tests in patients candidate to TRT (V, B) The presence of neurologic paraneoplastic syndromes that can be aggravated by immunotherapy must be ruled out (V, C) Full staging includes: CT scan with intravenous contrast of the chest/abdomen, MRI (preferred), or CT scan (with intravenous contrast) for brain imaging (III, A) 18F-FDG-PET/TC scan is recommended in localized disease to assist to thoracic radiotherapy (III, A). In patients with a solitary metastasis, its pathological confirmation is recommended (III, C) Bone marrow aspiration or biopsy is recommended if direct or indirect data of bone marrow infiltration (III, B) 8th edition of the TNM staging system according to the AJCC should be used (Table 1) (I, A). Combined use of TNM and VA classification is appropriate
Management of limited Stage I–IIA (T1–T2, N0, M0)	Surgery should be recommended in patients with clinical stages I and II (cT1-2N0) (III, B) Lobectomy with a systematic lymph-node dissection is the preferred surgical procedure after mediastinal staging (II, A) ChT and TRT (IV, A) concurrent (preferred) or sequentially (IV, A) should be recommended in patients with R0 pN1–pN2 or R1–R2 after surgery Patients with N0 disease should be recommended adjuvant chemotherapy (IV, A) SBRT (≥ 50 Gy) represents an alternative for patients with stage I–IIA SCLC with surgical contraindication or refusing surgery. After completion of SBRT patients should receive four cycles of adjuvant chemotherapy (III, A) PCI is not recommended in this subgroup of patients (II, E)
Management of limited-stage IIB–IIIC (T3–4, N0 M0; T1–4, N1–3, M0)	Patients should be treated with concurrent ChT and TRT (I, A) The recommended ChT is the combination of 4 cycles of cisplatin–etoposide (I, A). Carboplatin could replace cisplatin when contraindication (II, A) ChT dose reductions should be avoided, especially during the first two cycles of treatment (II, B) The use of G/GM-CSF is safe, when clinically indicated (II, B) 45 Gy with twice-daily fraction (I, A) or 60–70 Gy (II, A); with once-daily fraction are accepted treatments. Either of them should be administered concomitantly to systemic therapy (II, A) RT should be started as early as with the 1st or 2nd course of ChT (II, A) PCI (25 Gy in ten daily fractions) should be administered after CRT in patients without progression (I, A) Hippocampal avoidance PCI is an alternative option to PCI (II, B)
Management of extensive-stage (any T, any N, M1a, b, c): first-line treatment	The recommended first-line treatment is the use of platinum–etoposide + IO (I, A) Atezolizumab–carboplatin–etoposide 4 cycles followed by maintenance atezolizumab Durvalumab + carboplatin or cisplatin–etoposide 4 cycles followed by maintenance durvalumab If no candidate to receive IO, the recommended treatment is chemotherapy 4 cycles of cisplatin–etoposide (I, A). Carboplatin could replace cisplatin when contraindicated (I, B) Alternative regimens are cisplatin–irinotecan, carboplatin– irinotecan (II, B)
Management of extensive-stage (any T, any N, M1a, b, c): radiotherapy	Consolidative thoracic radiation to the residual tumor and lymph nodes (30 Gy/10 fractions) in selected patients who achieved a response to ChT is a treatment option (II, B) PCI (25 Gy) should be evaluated in patients with good PS who achieve a response (II, B) An alternative to PCI in patients without brain metastases on brain MRI after ChT is follow up with regular brain MRI omitting PCI (II, B) The benefit of adding PCI in patients receiving ChT–IO has yet to be determined (V, C)
Second-line treatment in ES-SCLC	Retreatment with platinum–etoposide is recommended for patients with sensitive relapse (platinum-free interval ≥ 3 months) (I, A) Single-agent topotecan is recommended for patients with refractory disease, resistant relapse, or in patients with sensitive relapse that are not candidates for platinum rechallenge (e.g., ECOG PS > 1, prior significant toxicity with doublet platinum-based ChT, or any other contraindication to receive platinum) (I, B) In this same situation, CAV (II, B), irinotecan (III, B) or weekly paclitaxel (III, C) are also reasonable treatment options Single-agent lurbinectedin is clinically active in relapsed SCLC, and it can be considered and recommended in patients with relapsed SCLC regardless of platinum-free interval (III, A). At the time of writing guideline document, lurbinectedin is FDA approved, has granted orphan drug designation by the EMA, but not authorized in Spain Single-agent PD-1 axis blockade is not generally recommended in unselected patients with relapsed SCLC (I, D)
Elderly and frail patients	In LS-SCLC, concurrent cCRT with modern technics could be a treatment option for fit and elderly patients (IV, B) Unfit patients ineligible for cCRT may be considered for sequential (II, C) For elderly ES-SCLC patients, carboplatin/etoposide is preferred than cisplatin/etoposide (I, B) In ES-SCLC, ChT–IO combination are recommended as first-line treatment (I, B) Shared decision process to indicate PCI over close surveillance is recommended in older patients with LS-SCLC Active CNS surveillance than PCI is preferred in older patients with ES-SCLC (I, A)
Follow-up	LS-SCLC: CT scan every 3 months the first year, every 6 months year 2–3 and after annually (V, C) ES-SCLC: CT scan every 2–3 months the first year, every 3 months year 2 and 3, every 6 months year 4–5 and then annually (V, C) MRI (preferred) or CT brain with contrast every 3 months during the first year, then every 6 months thereafter are recommended in patients who did not undergo PCI

The Acknowledgments and Conflict of interest sections were missing from this article and should have read as follows.

**Acknowledgments**

The authors thank Enriqueta Felip y Javier de Castro for their review and validation of the levels of evidence and grades of recommendation in this guideline.

**Conflict of interest**

RGC reports Advisory boards, Consultancy and Speaker honoraria from MSD, BMS, Roche, Boehringer Ingelheim, Pfizer, Novartis, AstraZeneca, Lilly, Takea and Amgen. IS reports Advisory Board from Roche, Novartis, Boehringer Ingelheim, Takeda and Sanofi; Speaker from Roche, MSD, Pfizer, BMS and AstraZeneca; Grant from Roche, Takeda, Pfizer, BMS and AstraZeneca; Non-financial Support from Member of GECP. EA reports Advisory Board, Speakerand Grant from Roche; Advisory Board and Speaker from Astra Zeneca and BMS; Advisory Board, Speaker and Non-financial Support from Takeda; Advisory Board from Lilly and Boehringer-Ingelheim; Speaker from MSD, Merck, Thermo Fisher Scientific and Guardant Health; Speaker and- Non-financial Support from Pfizer. AI reports Advisory Board and Speaker from Roche, AstraZeneca and Sanofi; Speaker from Takeda and BMS. OJJV reports Advisory Board and Speaker from BMS and Janssen; Speker from Roche; Advisory Board, Speaker and Other from AstraZeneca, Takeda and Janssen; Advisory Board from Lilly. NR reports Advisory Board, Speaker and- Other from MSD; Advisory Board and Speaker from AstraZeneca, Takeda, Amgen, Lilly, Sanofi, Roche and Janssen. JZ reports Speaker, Grant and Personal Feels from BMS and AstraZencea; Advisory Board and Personal Feels from Sanofi; Speaker from MSD; Grant and Personal Feels from Roche; Speaker and Personal Feels from Pfizer; Advisory Board from Novartis and Speaker from NanoString. MD reports Advisory Board and Speaker from AatraZeneca, Pfizer and Takeda; Speaker from BMS, MSD and Roche; Advisory Board from Janssen and Sanofi. TM and PCC have nothing to disclose.

The original article has been corrected.

